# Dr. Robert Hill Dee

**Published:** 1908-04

**Authors:** 


Dr. Robert Hill Dee, of Brockport, N. Y., died at his home January 26, 1908, of cerebral hemorrhage, aged 78 years. He graduated at the University of Buffalo in 1852, and was physician to the Six Nations Indians, Ontario, for thirty-five years; and for more than twenty years was coroner of Brant County, Ont.

